# Schaalia (Formerly Actinomyces) turicensis Infection Following Open Rotator Cuff Repair

**DOI:** 10.7759/cureus.34242

**Published:** 2023-01-26

**Authors:** John T Cronin, Brett W Richards, John G Skedros

**Affiliations:** 1 Shoulder & Elbow, Utah Orthopaedic Specialists, Salt Lake City, USA; 2 Department of Orthopaedics, University of Utah, Salt Lake City, USA

**Keywords:** irrigation and debridement, shoulder surgery, actinomyces turicensis, schaalia turicensis, infection, open rotator cuff repair

## Abstract

We report the case of a male in his early 70s who developed a deep infection after an open rotator cuff repair, with *Schaalia turicensis* as the only organism isolated from a surgical biopsy of the tendon remnants and phlegmatic/purulent material at the failed repair site. This species was originally within the genus *Actinomyces*. We report this case because it is the only one that we could locate where an infected open rotator cuff repair site grew *S. turicensis*. Our patient was not diabetic, did not smoke, and did not have other recent or concurrent infections. He had hypertension, hypothyroidism, depression, and a hyperactive bladder. Hence, he only had minor risk factors for infection. His postoperative shoulder infection was eradicated with surgical irrigation and debridement, and 6.5 weeks of primarily oral antibiotic treatment. We also review the literature on infections after any shoulder surgery where *Schaalia* or *Actinomyces* species were isolated.

## Introduction

Deep infections of the shoulder following an open rotator cuff repair (RCR) are relatively uncommon, with reported rates ranging from 0.3-1.9% [1-8]. The risk of infections after arthroscopic RCR is considered to be lower than open RCR with reported rates of less than 1% [8-10]. However, these studies used retrospective data and may have missed some infections after arthroscopic RCR in patients who sought follow-up care at other institutions [2]. Jensen and co-workers reported that while the rate of superficial infections is higher after open RCR, the rate of deep infection appears to be similar between open and arthroscopic RCR surgery [8]. The most common organisms isolated from open and arthroscopic RCR deep infections are *Cutibacterium acnes* (previously *Propionibacterium acnes*), coagulase-negative *Staphylococcus* species, and *Staphylococcus aureus* [1,2,8,10]. Other less common organisms isolated from infections following these procedures have also been previously described [3-7,11-14].

We report a unique case of deep shoulder infection following an open RCR where the only isolated organism was *Schaalia turicensis* (previously *Actinomyces turicensis*) [15,16]. This genus name honors Klaus P. Schaal who did extensive work on *Actinomyces* systematics [15]. Although some *Actinomyces* species, like *A. turicensis*, have been added to the *Schaalia* genus (now *S. turicensis*), the genus *Actinomyces* still exists and includes approximately 25-30 species [17,18].

A few previous reports of infections after open and arthroscopic RCR have reported the presence of an *Actinomyces* species; however, we could not locate any study that reported any of its exact species (including *A. turicensis*) [2,10,14]. *Schaalia *or *Actinomyces* species have been isolated in cultures from deep infections occurring after several types of shoulder surgeries including RCR, arthroplasty, and glenohumeral instability [10,14,18-21]. We are reporting this case to contribute to the literature describing unusual deep infections following RCR surgery and to highlight a rare organism and successful treatment regime.

## Case presentation

Our patient is a male in his early 70s (180 cm tall, 95 kg, and BMI 29) who was seen in our clinic with signs and symptoms of a deep shoulder infection nine weeks after one of us (JGS) performed an arthroscopic debridement of a degenerative labrum tear followed immediately with a mini-open RCR of the left shoulder. The patient had hypertension, hypothyroidism, depression, and a hyperactive bladder. He was not diabetic, did not smoke, never used illicit drugs, did not have recent dental work, and there was no evidence of other recent or current infections that might have locally or hematogenously seeded his RCR site [22]. His routine medications included benazepril, levothyroxine, sertraline, and mirabegron. The patient’s primary care provider had administered a corticosteroid injection in his left subacromial space five weeks before this left shoulder RCR. The RCR utilized transosseous sutures for a 2.5 cm minimally retracted full-thickness supraspinatus tendon tear.

The surgical site had been draining turbid serosanguinous fluid for nearly one week by the time he brought it to our attention. The surgical incision was five centimeters in length and was over the superior-lateral shoulder. The central two centimeters was mildly dehisced with a four cm diameter region of erythema. The patient was not taking antibiotics. Tissue cultures were obtained from within the dehisced location (without touching the skin and after wiping the skin margins with 70% alcohol swabs). The cultures were kept for 14 days, but no growth occurred. The patient was admitted to the hospital that same day and blood tests revealed a normal white blood cell count, an elevated erythrocyte sedimentation rate (ESR) (46mm/hr, normal = 0-20mm/hr), and an elevated C-reactive protein (CRP) level (2.7mg/dL, normal = 0-1.0mg/dL). He was then treated with IV vancomycin and IV Zosyn® (piperacillin-tazobactam), which were the first antibiotic doses given for this infection.

Arthroscopic and open surgical irrigation and debridement (I & D) were performed one day later. Surgical findings confirmed that the infection was deep, involving the repair site but without gross evidence of osteomyelitis. As discussed below, there was no evidence of the more extensive and typically chronic infection known as actinomycosis, which is a granulomatous infection characterized by the formation of tiny clumps called sulfur granules because of their yellow color [17]. Two intraoperative tissue cultures were taken of the tendon remnants and phlegmatic/purulent material at the failed repair site [17]. Six days later, these cultures grew *S. turicensis*. Identification was done using matrix-assisted laser desorption/ionization time-of-flight mass spectrometry (MALDI-TOF MS) [23].

Two days after surgery, an infectious disease consultant adjusted the antibiotic treatment to 600mg linezolid and 500mg ciprofloxacin for broad coverage; both taken orally twice a day for two weeks. At 2.5 weeks after surgery, the infectious disease consultant then considered the infection to be monomicrobial and caused by *S. turicensis*, and therefore continued antibiotic treatment with oral amoxicillin (one gram three times per day). Three weeks after the I & D surgery, the patient’s ESR and CRP were normal.

At the final follow-up at 14 months after the I & D surgery, he was tolerating the weakness in his shoulder and his pain remained negligible because he limited attempts at upward reaching, reflecting the fact that the tear was larger and not reparable. No additional surgery was planned because he felt that his outcome was very good.

## Discussion

Species in the *Schaalia* and *Actinomyces* genera are typically slow-growing, microaerophilic-to-facultative anaerobic, gram-positive, filamentous, branching bacilli that are part of the normal commensal flora in the mouth, pharynx, gastrointestinal tract, and female urogenital tract [17,24]. *S. turicensis* is generally seen in the genital and urinary tracts, and in infections related to skin and intrauterine devices (IUDs) [24-29]. As shown in Table 1, only two previous studies have reported *Actinomyces* in cultured organisms from deep infections after RCRs [10,14]. These studies, however, identified only the *Actinomyces* genus, making our report of *S. turicensis* in a deep shoulder infection unique (including the prior nomenclature, *A. turicensis*). It should be noted, however, that cases similar to our patient may be underreported due to the prevalence of culture-negative infections following arthroscopic and open RCR. Hughes et al. reported a culture-negative infection rate in five of 20 patients (25%) who had grossly confirmed deep infections after open or arthroscopic RCR (i.e., all 20 patients had surgical debridement and cultures, but five showed no growth) [12]. We extended our literature search to see if we could find *S. turicensis* in any other types of shoulder conditions and surgeries. We found a few cases of *Actinomyces* species reported as the potential causative organism of postoperative shoulder infections, but not *A. turicensis*. The organisms that we found included *A. odontolyticus*, *A. meyeri*, *A. neuii*, and one *Actinomyces* species that was not further identified [18-21].

**Table 1:** Deep Infection Following Arthroscopic and Open Rotator Cuff Repair *Coagulase-negative Staphylococci include, but are not limited to, *S. epidermidis*, *S. saprophyticus*, and *S. hominis*. mono: monomicrobial, sp.: species; *P. acnes*: *Propionibacterium acnes; C. Acnes: Cutibacterium acnes* This table was first published in the World Journal of Orthopaedics, vol. 8 (8), by Atesok et al. [2], pg. 612-618, and is licensed under the Creative Commons Attribution Non-Commercial [CC BY-NC 4.0] license. This allows for the non-commercial distribution and modification of this work, and licensing of derivative works on different terms, provided the original work is properly cited. See: http://creativecommons.org/licenses/by-nc/4.0/

Reference	Surgery Type	No. Patients (isolates)	No. of isolated organisms	P. acnes (C. acnes)	S. aureus	Coagulase-negative staphylococci*	Other organisms
Mansat et al. 1997 [4]	Not specified	2 (monomicrobial)	2	0	1	0	*Bacillus* sp.
Settecerri et al. 1999 [6]	Not specified	16 (15 mono, 1 polymicrobial)	17	7	4	5	1 (*Peptostreptococcus magnus*)
Mirzayan et al. 2000 [5]	Open	13 (7 mono, 3 polymicrobial, 3 negative)	15	3	5	5	2 (Diptheroids and Streptococcal sp.)
Herrera et al. 2002 [3]	Mini-open	7 (6 mono, 1 polymicrobial)	9	6	1	1	1 (*Pseudomonas* sp.)
Kwon et al. 2005 [7]	Open, Mini-open	14 (11 mono, 3 polymicrobial)	19	7	4	6	2 (*Proteus mirabilis* and* Enterococcus faecalis*)
Athwal et al. 2007 [1]	Open, Mini-open	38 (39 shoulders: 33 mono, 6 polymicrobial)	45	20	8	12	5 (2 *Corynebacterium* sp., *Peptostreptococcus magnus*,* Bacillus* sp., *Streptococcus viridans*)
Vopat et al. 2016 [11]	Open, Mini-open, Arthroscopic	14 (9 mono, 5 negative)	9	4	1	4	NA
Atesok et al. 2017 [2]	Arthroscopic	10 (7 mono, 2 polymicrobial, 1 negative)	11	2	5	3	1 (*Phaeoacremonium parasiticum*)
Hughes et al. 2017 [12]	Open, Mini-open, Arthroscopic	20 (likely mono, 5 negative)	15	6	7	1	1 (*Clostridium* sp.)
Pauzenberger et al. 2017 [10]	Arthroscopic	28 (likely mono, 5 negative)	23	8	2	12	1 (*Actinomyces *sp.)
Jenssen et al. 2018 [13]	Arthroscopic	11 (5 mono, 6 polymicrobial)	19	10	0	9	NA
Frank et al. 2020 [14]­	Arthroscopic	30 (likely mono, 5 negative)	25	9	3	12	1 (*Actinomyces* sp.)
Current Study	Mini-open	1 (monomicrobial)	1	0	0	0	1 (*Schaalia turicensis,* formerly *A. turicensis*)

In contrast to the postoperative infection that occurred in our patient and in the two cases with *Actinomyces *species listed in Table 1, actinomycosis is a condition that is also caused by *Actinomyces* species but manifests very differently from postoperative, more localized, acute to sub-acute infections. Actinomycosis is typically chronic and is characterized by firm-to-hard mass-type (“clump”) lesions of the branching filamentous bacilli that characterize *Actinomyces*, with abscesses, draining sinuses, and tissue fibrosis [24,30]. Actinomycosis progresses from a site of inoculation to a typically more extensive infection with intense fibrosis of tissue [31]. Notably, our patient did not have actinomycosis (though it is conceivable that he could have developed this if left untreated) [32]. This fact is important to emphasize because actinomycosis often requires prolonged antibiotic treatment (6-12 months) [24,33]. Nevertheless, it is important to consider actinomycosis, caused by *Actinomyces* species or similar *Schaalia* species, as the etiology of our patient’s infection because actinomycosis can present as a primary cutaneous infection in healing surgical scars [34]. However, as mentioned in the Case Presentation section, characteristics of primary cutaneous actinomycosis were not detected by gross visual and manual palpation examination of our patient’s incision and subcutaneous tissues, and *S. turicensis* grew only from deep wound cultures and not from the superficial wound culture. These factors demonstrate that our patient did not have actinomycosis. Consequently, relatively prolonged antibiotic treatment was not needed to eradicate his infection. Similar relatively short courses of antibiotics have also been reported as being successful in the treatment of some cases of actinomycosis [35].

Previous studies have reported that *Actinomyces* species do not exist freely in nature but are commensals and normal inhabitants in humans [17,30]. Hence, humans are the natural reservoir of these *Actinomyces* and *Schaalia* species that cause actinomycosis and more localized infections (as seen in our patient) [30]. It is possible that the *S. turicensis* that was cultured from our patient’s deep shoulder tissue was present in his skin microbiome and was introduced during surgery [24]. Indirect support for this idea can be gleaned from the study of Herrera et al., who reported on a series of 360 patients (mean age = 55 years; range 40-64 years) [3]. They found that, similar to our case, the patients who underwent mini-open rotator cuff repairs immediately after limited arthroscopy were the most likely to develop postoperative infections. In fact, *P. acnes* (now *C. acnes*) was present in six of seven patients (86%) with infections (none were associated with *Actinomyces *or *Schaalia* species). In view of this high incidence, they hypothesized that fluid extravasation during arthroscopy decreased the efficacy of the preoperative preparation, which allowed the organism easy access inside the shoulder once the incision has been made for the mini-open portion of the procedure. They dropped their infection rate to nil after instituting a second Betadine® paint prep and changing the surgeon’s gloves between the arthroscopic and open procedures. It is theoretically possible that arthroscopic-fluid-related inoculation of *S. turicensis* occurred during our patient’s surgery [36].

The close proximity (five weeks) of our patient’s subacromial corticosteroid injection to his open RCR may have increased the risk of postoperative infection. This supposition is based on anecdotal and retrospective observations [7,37]. However, a study of 12,060 patients who had a shoulder corticosteroid injection within one year of arthroscopic RCR on the same shoulder found that the risk of infection was significantly increased only when the injection is given within one month of the surgery [38]. While this suggests that our patient’s antecedent corticosteroid injection did not increase his risk of a deep postoperative infection, this still remains a possibility because our patient had an open RCR. Additional study of a similarly large sample of patients who had open RCR and antecedent corticosteroid injections is needed to assess this possibility. Other risk factors identified by Forsythe et al. included male sex, smoking, obesity, and diabetes [38]. Our patient only had the "male sex" risk factor. 

The possibility that *S. turicensis *was a contaminant and not the actual causal organism should be considered. Also, a culture obtained from the superficial aspect of the wound 1.5 days before our patient’s debridement surgery did not grow anything even though antibiotics (oral or IV) had not been given and the culture was held for 14 days. Perhaps growth would have occurred had cultures been kept for 21 days; this supposition is based on reports that growth of *Actinomyces* species in rare instances can occur between 14 to 20 days of culture [17].

Our patient received IV antibiotics for three days during his hospitalization for the acute infection treatment. Thereafter, he was treated only with oral antibiotics, which is typically sufficient for bone and joint infections that do not include prosthetic implants [39]. The final four weeks of his antibiotic treatment were with 1000mg of amoxicillin three times per day. This antibiotic choice reflects the fact that *Actinomyces* and *Schaalia* species are widely susceptible to beta-lactam antibiotics [40]. Consequently, we did not perform antibiotic sensitivities.

A study of clinical isolates from various sources (none of which were from the shoulder region) showed that common co-isolates of *S. turicensis* include *S. anginosis* (a gastrointestinal clade), *Escherichia coli*, *Enterococcus*, and *Aerococcus urinae* [25]. Co-isolates were not found in our case. Nevertheless, it is also possible that our patient had a polymicrobial infection, but the additional organism(s) did not grow because three doses each of Zosyn and vancomycin were given prior to the I & D surgery. However, in contrast to other anaerobic infections, which may exist as part of a polymicrobial infection, infections due to *Actinomyces* species are monomicrobial in most cases [22].

## Conclusions

There are no prior reports of *S. turicensis* (formerly *A. turicensis*) as an organism isolated from deep tissue culture of an infected rotator cuff repair. Our septuagenarian patient had the *S. turicensis* infection after his rotator cuff tear even though he was not diabetic, did not smoke, and did not have other recent or concurrent infections. He had hypertension, hypothyroidism, depression, and a hyperactive bladder. Hence, he only had minor risk factors for infection that included a mildly elevated BMI and his age. His infection was successfully treated with one surgical debridement surgery, three days of IV antibiotics, and six weeks of oral antibiotics. *S. turicensis* can be treated with beta-lactam antibiotics, which were also primarily used in our patient.
